# Considerations for MRI study design and implementation in pediatric and clinical populations

**DOI:** 10.1016/j.dcn.2015.12.005

**Published:** 2015-12-17

**Authors:** Deanna J. Greene, Kevin J. Black, Bradley L. Schlaggar

**Affiliations:** a Department of Psychiatry, Washington University School of Medicine, St. Louis, MO, United States; b Department of Radiology, Washington University School of Medicine, St. Louis, MO, United States; c Department of Neurology, Washington University School of Medicine, St. Louis, MO, United States; d Department of Neuroscience, Washington University School of Medicine, St. Louis, MO, United States; e Department of Pediatrics, Washington University School of Medicine, St. Louis, MO, United States

**Keywords:** Neuroimaging, Development, Neuropsychiatric disorders, Inclusion/exclusion criteria, Mock scanner, Motion artifact, Tourette syndrome

## Abstract

Human neuroimaging, specifically magnetic resonance imaging (MRI), is being used with increasing popularity to study brain structure and function in development and disease. When applying these methods to developmental and clinical populations, careful consideration must be taken with regard to study design and implementation. In this article, we discuss two major considerations particularly pertinent to brain research in special populations. First, we discuss considerations for subject selection and characterization, including issues related to comorbid conditions, medication status, and clinical assessment. Second, we discuss methods and considerations for acquisition of adequate, useable MRI data. Given that children and patients may experience anxiety with the scanner environment, preventing participation, and that they have a higher risk of motion artifact, resulting in data loss, successful subject compliance and data acquisition are not trivial tasks. We conclude that, as researchers, we must consider a number of issues when using neuroimaging tools to study children and patients, and we should thoughtfully justify our choices of methods and study design.

## 1 Introduction

With the rising use of human neuroimaging techniques to answer research questions of developmental and clinical significance, there is increasing need for a comprehensive understanding of strategies for collecting imaging data in special populations. While investigators continue to develop new approaches for acquiring quality data, the field would benefit from further refinements and updated strategies to optimize the cost:benefit ratio of our imaging studies. Here, we discuss two overarching considerations for MRI data acquisition in pediatric and clinical populations: (1) subject selection and characterization, and (2) methods for acquiring adequate, high-quality imaging data. These topics often prompt debate or criticism from reviewers for journals and funding agencies. Thus, it is important that these key issues be well understood.

In this article, we refer often to our own experience collecting data from children with Tourette syndrome (TS), which exemplifies many of the key issues researchers face in acquiring data from pediatric and clinical populations. TS is a neuropsychiatric disorder with childhood onset. Thus, many of the issues that arise in TS research are similar to those in studies of typical development as well as other childhood disorders, such as autism and attention deficit hyperactivity disorder (ADHD). TS is characterized by motor and vocal tics, which are brief, unwanted movements or sounds (Leckman et al., 2006, Black et al., 2014); common tics include exaggerated eye blinking, head jerking, sniffing, and throat clearing. Therefore, special considerations are required with respect to motion during data acquisition, as is the case in studies of child populations in general as well as other movement disorders, including Parkinson disease and dystonia. Also, like many neuropsychiatric disorders, TS has a high comorbidity burden, with particularly high rates of comorbid ADHD and obsessive compulsive disorder (OCD) (Freeman et al., 2000). Moreover, patients can be treated with behavior therapy (e.g., Piacentini et al., 2010) or with a variety of psychoactive medications, including antipsychotics, centrally acting adrenergic agents, and SSRIs (Black et al., 2014, Eddy et al., 2011). Thus, issues that must be considered with respect to comorbid conditions and medication status in studies of TS also apply to research in other clinical populations. Further, while we focus here on MRI data collection in typical and atypical development, the issues discussed apply to studies in a wide range of clinical populations and across the lifespan.

## 2 Considerations for subject selection and characterization

An important, early step for neuroimaging research is appropriate study sample selection. While sample selection may seem trivial initially (e.g., research aimed at studying TS will include children with a diagnosis of TS), there are important issues to consider when determining study inclusion/exclusion criteria. One might think that the best approach to studying any neuropsychiatric population is to restrict subject selection to a “clean” or “pure” sample with no comorbid conditions or current medications. While there are scenarios in which this approach is appropriate, it is underappreciated that such an approach can raise as many concerns as it solves. Here, we discuss these concerns, offer our opinions, and describe strategies implemented in our own research studies.

### 2.1 What is the ultimate goal of your research?

Investigators must consider the ultimate purpose of their research program. When designing an experiment, researchers regularly evaluate aspects of study design (including subject inclusion/exclusion criteria) with respect to the particular experimental question at hand. We argue that in addition to considerations related to the specific experiment, investigators ought to reflect on the overall motivation behind that study and the eventual goal in the context of the greater research program. In other words, what does the “Significance” or “Impact” section of the grant supporting the work claim? Is the aim of the work to understand the neural correlates of a particular disorder as a whole rather than a single symptom? Is the overarching objective to inform future discovery of better treatment targets or clinical care? In our own studies and often in other laboratories, the answer to these questions is “yes.” In such cases, results that are generalizable to most individuals with the disorder are most desirable. Indeed, there is research aimed at understanding and/or targeting one specific symptom (e.g., tics, impulsivity) of a complex disorder (e.g., TS, ADHD). For these studies, it may be more appropriate to take a restricted approach to subject inclusion in order to isolate the symptom under study. Yet, researchers must understand the important distinction between studying the mechanisms of a specific *symptom* and studying the mechanisms of a *disorder*. Typically, a disorder is characterized by many types of symptoms—thus, the appellation “syndrome”. TS, for example, while defined by the involvement of motor/vocal tics, most often presents clinically with any one of a number of cognitive and behavioral symptoms (Freeman et al., 2000, Martino et al., 2013). Therefore, any study of TS must consider whether the goal is to investigate tics in isolation or the clinical presentation typical of the majority of patients. If only a small percentage of individuals with the disorder have no other known neuropsychiatric problems, as is the case with TS, research limited to understanding that small fraction of individuals may have limited applicability, at least initially. Even a study focused on the neural mechanisms of tics (or treating tics) in isolation with a clear justification for studying a pure and unmedicated sample is but a first step toward understanding TS as a whole. Such a study will require follow-up research to test whether or not the findings generalize to those patients with more typical clinical presentation of the disorder (i.e., with comorbid conditions, medication history). Moreover, if the translational significance of the research is to inform future discovery of treatments, researchers ought to consider whether the population studied includes those individuals who are most likely to seek clinical care (Gilbert and Buncher, 2005). Thus, when the ultimate goal is to understand or treat the complex disorder, we argue that ignoring those patients with the most typical clinical presentations will limit the clinical applicability of the research, impeding its ultimate purpose. In other words, we prefer to collect an ecologically valid sample for most neuroimaging (and non-neuroimaging) studies. There are two approaches one can take to achieve such ecological validity: (1) first study a pure sample, and then conduct follow up research to test the generalizability of the results, or (2) study a larger heterogeneous sample, and conduct subgroup analyses to test the specificity of the results. Some may opt for the former option, but we opt for the latter. Either way, an ecologically valid sample will ultimately need to be studied in order to obtain truly translational results.

We recognize that ecological validity may not be entirely feasible for some neuroimaging research. For example, many MRI studies in autism spectrum disorders (ASD) include only patients with high-functioning ASD. Lower functioning ASD patients may not be able to lie inside the scanner without sedation or may not be able to understand or comply with task instructions. Therefore, it is not practical to include these lower functioning children with ASD in an fMRI study of working memory, for example. In such cases, it is defensible to exclude subjects who could not practically participate, but limitations in generalizability must be acknowledged. When feasibility is not a limiting factor, however, we recommend striving for ecological validity. In our own studies, we aim to place the burden of heterogeneity on the analysis, not on narrowly focused recruitment, allowing for data collection that best captures the real-world population. Often, this strategy will necessitate large sample sizes in order to capture the heterogeneity, i.e., including both treated and untreated patients, and those with and without diagnosed comorbid conditions. In the following subsections, we discuss the less commonly considered issues with respect to comorbid conditions and medication status that have informed our decisions for subject selection, leading to our preference for ecologically valid samples in most of our neuroimaging studies.

### 2.2 Considering comorbid conditions

Comorbidities are quite common, and for some disorders the presence of comorbid conditions is the rule rather than the exception. TS is a prime example, as only 10% of individuals with TS have no other known comorbidities (Freeman et al., 2000). The most common comorbid conditions are ADHD and anxiety, most frequently OCD. In fact, 50–60% of children with TS also have ADHD and 30–40% also have OCD, and so TS, OCD, and ADHD are often discussed as an interconnected triad (Kurlan, 2010). Therefore, if one is interested in studying TS as a condition, these common comorbidities must be considered. Selecting only those TS patients with no comorbid conditions will be logistically difficult from a recruitment standpoint and will usually limit the generalizability of the results. TS is not alone in this regard; mood disorders, anxiety, and sleep disturbances often co-occur (Vazquez et al., 2014, Chorney et al., 2008), ADHD is frequently comorbid with conduct disorder, oppositional defiant disorder, dyslexia, and learning disabilities (Pliszka, 1998, Germano et al., 2010), and substance abuse is highly comorbid with mood and anxiety disorders (Conway et al., 2006). Thus, these principles apply to many studies of neuropsychiatric disorders.

Reviewers of grants and research manuscripts often request that inclusion and exclusion criteria result in “clean” or “pure” samples of patient populations, meaning excluding all comorbid conditions. It has become easy to make such a request reflexively, and colleagues often support it as reasonable. We argue that the issue is more complicated, and requires careful consideration of the consequences and limitations that arise when using pure vs. heterogeneous samples. One concern is that defining a truly pure sample is not straightforward. The recent release of DSM-5 highlights the fact that diagnostic criteria constitute a moving target. For instance, changes in the diagnostic criteria for TS from DSM-IV to DSM-IV-TR to DSM-5 included changes in diagnostic threshold and symptom duration, such that a child may meet diagnostic criteria under one of these definitions but not the others. Even when the criteria do not change, symptoms change over time within an individual, especially when considering developmental populations. For example, a 7 year old child with “pure” TS at the time of study may 3 years later be a 10 year old child with TS, OCD, and a single depressive episode, posing the question of whether this child was ever really a case of pure TS. A similar concern applies retrospectively, since both patient recall and clinical judgment are fallible (Kendler et al., 1993, Kendler et al., 1999). Moreover, family history of neuropsychiatric illness provides information about liability in the offspring, yet family neuropsychiatric history is almost never assessed in neuroimaging studies. Additionally, patients often have subclinical symptoms; a patient meeting diagnostic criteria for TS but not for OCD or ADHD may nevertheless have nontrivial compulsions or hyperactivity. Altogether, it is problematic to claim definitively that a group of patients has one and only one neuropsychiatric condition.

The reflexive insistence on pure samples also ignores important realities that may have unintended consequences for the validity of a study. Depending on the disorder, a pure sample may lead researchers to examine brain function in exceptional cases rather than typical cases. In TS, in which the clinical presentation involves significant heterogeneity and high comorbidity, a pure sample will consist of the more rare cases. Thus, even if it were possible to obtain a truly clean sample, studying the 10% of TS patients with no known comorbid conditions (Freeman et al., 2000) and unmeasured family history likely will not yield results that are generalizable to most patients with TS. Similarly, a study of substance abuse that excludes the ∼40% of patients with comorbid mood disorders and the ∼30% with comorbid anxiety disorders (Conway et al., 2006) will not best represent the clinical population. In these instances, the most ecologically valid sample will include comorbidities, and the implications of limiting the study population to the rare cases must be acknowledged. In addition, TS is exemplary of disorders in which more severe symptoms tend to be associated with more comorbidity. Consequently, an unintended byproduct of including only pure TS cases will be to limit the sample to those with milder symptoms. The patients that researchers and clinicians are most interested in helping are not the ones with the lowest symptomatic burden. Thus, in disorders similar to TS, we recommend taking an inclusive approach for most studies rather than limiting the subject pool to the least affected patients. As there may be disorders in which symptom severity is not related to comorbidity burden, investigators must understand the factors affecting their population of interest and carefully consider such factors when striving to study an ecologically valid sample. Indeed, as we mentioned previously, some studies will be aimed at identifying the mechanisms of a specific symptom (e.g., tics, addiction) in which more restrictive inclusion criteria can be acceptable. However, when the greater purpose of a body of work is to move toward clinical utility, the results ultimately will need to be tested for generalizability to a “real world” sample.

Pure samples are often advocated as a way of controlling for confounding variables. However, controlling for these seemingly “confounding variables” by excluding comorbidities is not as straightforward as it may seem. For one, commonly comorbid conditions are not necessarily *confounding*, most obviously when a large proportion of patients share that comorbidity. Confounds suggest additive effects that can be removed or accounted for with methods like regression and covariance. Rather, common comorbidities may be more aptly termed *interacting* variables, as they interact in complex ways. Therefore, excluding for comorbid conditions will ignore the complex interactions that are often integral to the disorder. Examples of these complex interactions include ADHD in TS, or intellectual disability in autism. In addition, it has been argued that the term “comorbidity” can reflect a limitation of the diagnostic system, in which the “real disease” produces symptoms that span several current diagnostic categories. For instance, Huntington disease is caused by an abnormality in a single gene, but can cause chorea, dystonia, rigidity, depression, personality changes, and dementia in different people or across time in the same person. This idea underscores the importance of embracing the complexity that is the reality of neuropsychiatric illness. Thus, just as studies with heterogeneous samples are expected to acknowledge limitations, studies with pure samples must acknowledge their limitations as well, particularly with respect to the complexity of the disorder.

Though consideration of comorbidity will likely yield a complex sample, not only will this complexity more validly represent the true population, it will also be a fruitful avenue of study. High comorbidity of certain disorders brings up the question of whether the underlying brain mechanisms are overlapping or separable. While there are certainly cases of TS without other diagnoses, the large number of individuals with TS, OCD, and ADHD suggests the possibility that the underlying neurobiological mechanisms may not fit neatly within diagnostic lines. In fact, application of latent class analysis has provided evidence to suggest some overlap, identifying multiple classes, including a TS + OCD class and a highly heritable TS + OCD + ADHD class (Grados et al., 2008). Similarly, an analysis of children with ADHD and autism identified classes of ADHD alone and ADHD + autism, but not autism alone (van der Meer et al., 2012). Thus, studies aimed at investigating the overlapping and distinct neural correlates of these classes are greatly needed. Even within a diagnosis, studies aimed at understanding the brain mechanisms underlying different collections of symptoms would push the field forward immensely. One interesting finding to come out of an inclusive study design in adults with TS found that three clinically-defined subgroups showed reduced cortical thickness in different brain regions (Worbe et al., 2010). Patients with simple tics had cortical thinning in primary motor regions; patients with simple and complex tics had cortical thinning extending from primary motor regions to premotor, parietal, and prefrontal regions; and patients with tics and obsessive–compulsive symptoms had cortical thinning in the anterior cingulate cortex. Thus, including heterogeneous subjects and conducting subgroup analyses allowed for the interrogation of specific features relating to particular aspects of the disorder. Furthermore, treating subjects with a mixture of symptoms as a homogeneous group – whether mixing tics, obsessions, and compulsions, or mixing different types of tics – can obscure findings and may be responsible for inconsistencies in the literature (Greene et al., 2013). In fact, clustering methods and factor analysis of TS symptoms have identified subgroups even within a so-called pure TS group (McGuire et al., 2013, Cavanna et al., 2011). Additionally, there is recent evidence that clinical symptoms are not the only means by which to identify meaningful subgroups. Behavioral data measuring multiple cognitive functions as well as fMRI data can be used to identify behavior-based and imaging-based subgroups of children with ADHD, and even subgroups of typically developing children (Fair et al., 2012, Costa Dias et al., 2015). Thus, heterogeneous samples can be a virtue for many research questions, and can be presented as such in grants and manuscripts.

We also argue that subjects in neuroimaging studies should be characterized carefully clinically with reasonably deep phenotyping. For patients and controls alike, self-report of the presence or absence of a diagnosis is frequently inaccurate (King, 1997). Semi-structured clinical interviews greatly increase diagnostic reliability and provide more accurate and comprehensive assessments of clinical diagnoses. Clinical measures of current symptom severity (questionnaires or expert rating scales) can supplement – or, depending on the research aims, even replace – diagnostic interviews. Collecting dimensional clinical and behavioral data in addition to MRI data is crucial for improving the interpretability of study results. Studies aimed at investigating a particular neuropsychiatric disorder should include dimensional assessments of features that define the disorder as well as assessments of features that commonly accompany the disorder. For example, our studies of TS include measures of tic severity (e.g., Yale Global Tic Severity Scale; Leckman et al., 1989), as well as OCD severity (e.g., Children's Yale-Brown Obsessive Compulsive Scale; Scahill et al., 1997), ADHD symptoms (e.g., Conners’ Rating Scale; Conners et al., 1998), and anxiety (e.g., Multidimensional Anxiety Scale for Children; March et al., 1997), in addition to demographic information, behavioral measures of executive function, and general intelligence (Church et al., 2009a, Church et al., 2009b, Stewart et al., 2015, Greene et al., 2015, Greene et al., in press). Assessments that capture a range of sub-clinical and clinical symptoms are ideal, as subjects in a comparison control group may also exhibit symptoms below a clinical severity threshold. Similarly, behavioral measures of cognitive, motor, or sensory functions can be administered to both patient and control groups, allowing for dimensional comparisons. Collecting such data will allow for careful characterization of all subjects, and will enable analyses investigating the relationships between brain imaging data and clinical and behavioral features. Additionally, for the most complete and accurate assessments, collecting data from multiple informants is ideal. Given individual biases and the variability in how subjects or caretakers respond, relying on measures completed by a single informant may not be sufficient. Thus, whenever possible, we encourage the use of multiple informants (e.g., parent/guardian and teacher for children, self and close friend or relative for adults), either for questionnaires or clinical interviews with a trained professional. Some study questions may even require clinical consensus diagnosis based on all available data (Bryant et al., 1990). We recognize that such an intensive evaluation of all subjects is not always feasible, and the extent of phenotyping needed will depend on a study's specific aims. Admittedly, some of our previous studies lacked multi-informant assessment. However, our future studies will strive for a more complete approach and we encourage others to do the same.

### 2.3 Considering medication status

Patients with neuropsychiatric disorders are commonly treated with psychoactive medications. There are several issues to consider when including or excluding subjects due to past or present medication use, similar to the issues discussed regarding comorbidities. Often researchers assume that an unmedicated sample is a preferable sample. This assumption can lead to reflexive demands by reviewers for “medication-free” samples or by investigators themselves for unmedicated subjects. However, we argue that this assumption is not necessarily valid. While researchers readily point to issues associated with medicated subjects, a number of concerns also arise when excluding medications, and these concerns are less commonly considered. We do not mean to diminish the concerns that arise when including patients taking medications. Rather, we aim to enumerate the rarely discussed disadvantages of excluding medicated patients. These issues must be weighed against the better-known issues associated with including medicated patients, and adequately addressed in grant applications, grant reviews, and manuscripts.

One major issue is that the definition of “medication-free” is used variably. The term can refer to patients who discontinue their current medications during the study and for a washout period before; it may refer to patients with a past history of medication use, but who are not currently taking medications; or it can refer to patients who have never taken any psychoactive medications at all. The common practice of treating all of these “medication-free” subjects as equivalent is problematic. It is problematic to assume, for example, that the brain of a chronically medicated subject who does not take his/her medications the day of the scan is the same as the brain of a subject who has never taken a medication. Similarly, one ought not assume that the brain of a subject who is currently unmedicated, but has a history of medication use, is the same as the brain of a subject with no medication history (Robertson, 1996). In our experience, this issue is not regularly considered and subjects who have never taken medications, those with medication history who are currently unmedicated, and those who are currently medicated but discontinue medications for the experiment, are treated the same and deemed an unmedicated, “clean” sample. However, this unmedicated sample with hidden liabilities is not necessarily cleaner than a medicated sample in which the liabilities are known and taken into account.

Even when selecting a specific operational definition of medication-free, one must understand the potential unintended consequences. Excluding subjects with current medication use can introduce practical and scientific issues. For one, depending on the population under study, it can be difficult to recruit a large enough sample of unmedicated subjects to achieve sufficient power for neuroimaging research. Certain disorders necessitate medication treatment for the majority of patients, and many of those who are unmedicated may not be fit to participate in research (e.g., schizophrenia). In addition, certain recruitment sources do not always easily reach unmedicated patients. For example, recruitment via medical clinics will be limited by the patients seen at the clinic, who will often be medicated. Another issue to consider, again, is ecological validity. When the goal of a research program is to ultimately lead to novel targets for treatment and clinical care, a study sample that includes those patients who would hope to benefit from such treatment and care (who will likely have a medication history) is beneficial. Excluding subjects who are on medications may also produce unrecognized cohort effects. For example, such exclusion may bias the sample toward “healthier” patients, or in the case of pediatric patients, may select for patients whose parents have an aversion to medical therapy. Of course, any such effects can vary based on the study setting and population, but in the face of such cohort effects, further study will be needed to ensure that the results generalize to those patients who actively seek treatment.

Another point of consideration is that investigators must be aware of subjects’ treatment history. Patients who are not currently medicated may have a history of medication use, and that past medication use likely affected the brain's development. Further, patients who have never taken medications during their lifetime may have sought other forms of treatment, such as cognitive behavioral therapy, which also likely affected the brain's development. Thus, their brains are not treatment-naïve. Even patients who have not sought formal treatment, but have developed their own strategies for coping or improving symptoms have likely experienced brain changes. Therefore, a truly treatment-naïve sample without any previous alterations in brain function would be difficult to obtain and would likely not be representative of the general patient population.

Discontinuing current medications is another approach that comes with its own set of unintended consequences. One argument for this approach is to study a patient population in a “natural” or “clean” state. However, the effects of temporarily discontinuing medications for the study visit may interact with the subject's cognitive or affective state during the scan. Consider a child with ADHD who has been taking a stimulant for years and has experienced several benefits. This child is typically on the stimulant during school and in other settings when he/she is required to do attention-demanding tasks. Thus, he/she is now accustomed to doing such tasks while the stimulant is in effect. If the child is then taken off the medication, placed in the MRI scanner, and asked to perform attention-demanding tasks, he/she may be keenly aware of the resulting performance decrement and become frustrated or angry. The child may also approach the task with different cognitive strategies than would a never-medicated child. Thus, researchers should recognize the potential adverse effects this experimental maneuver can have on the subject's brain activity, and weigh such effects against those of not discontinuing the medication. Depending on the specific study aims, one option may be better than the other. Requiring medication discontinuation can also introduce selection bias. In our experiences, those patients with more severe symptoms who depend on medications sometimes choose not to participate if there is a requirement to discontinue their current medications even briefly, also reducing the generalizability of the results. Additionally, withdrawal of some medications causes biological changes that may be more obvious than the effect of the medication itself (e.g., alcohol withdrawal), or may be more permanent (e.g., tardive dyskinesia). Moreover, there are likely still brain effects of being chronically treated even if there has been temporary withdrawal (e.g., all antidepressant medications exert effects over a period of weeks). Finally, many medicines (e.g. fluoxetine or primidone) have long effective half-lives, making brief medication withdrawals futile. The point again is that withdrawing a medication may not be the same as not taking it in the first place. We recognize that there are circumstances in which medications have been shown to “normalize” brain measures (e.g., stimulants in ADHD, Schweren et al., 2013) and by removing the medication, differences between patients and controls can be more readily measured. Thus, the effects of discontinuing medications ought to be considered regarding the specific characteristics of the class(es) of medications and population under study.

Discontinuing medications for the purposes of research raises several ethical issues. For clinical trials investigating the efficacy of a medication, the risk of drug interactions can be an acceptable motivation for discontinuing current medications, though even then one must consider problematic consequences (Gilbert and Buncher, 2005). For other research, however, the ethical argument for discontinuing many psychotropic medications is weaker. Abrupt discontinuation sometimes will cause adverse side effects, from SSRI withdrawal syndrome (Schatzberg et al., 2006) to elevated seizure risk from stopping sedatives. In addition, withdrawing an effective medication will likely worsen symptoms, and this worsening may last a considerable period of time for medications that require a long weaning period. Issues to consider when deciding whether or not to discontinue medications include the subject's ability to give informed consent (e.g., the child's parent/guardian must provide informed consent—the child can only provide assent), whether discontinuation will cause problems beyond temporary cessation of the medication benefits, the extent of benefit the medication is providing, and any side effects of the medication. These issues will vary depending on the study population and the class of medication. For example, a study requiring discontinuation of antidepressants in adolescents with depression must consider the potential dangers of worsening symptoms as well as potential discontinuation side effects that may require a slow weaning period. On the other hand, discontinuing stimulants for a study of children with ADHD when the stimulant is the sole medication may be less problematic, as stimulants have a faster washout period and withholding them for one or several days does not pose medical concerns other than the worsening of ADHD symptoms. Thus, while there are circumstances in which taking children off their medications for the purposes of research may be acceptable, we argue for the justification to be thoughtful, clear, compelling, and well communicated. Overall, potential ethical concerns need to be considered and weighed for stopping each class of medication and for each disorder under study.

We recognize that if an fMRI study shows differences in brain activation between a patient group, all of whom are taking medications, and a control group, none of whom are taking medications, the neuropsychiatric condition under study and medications are confounded (though this extreme case is highly unlikely). Most investigators do not want to publish a finding that may be entirely attributable to medication-induced brain changes. At the same time, we have been arguing that an ecologically valid sample is beneficial in order to generalize research results to those patients with significant burden. Thus, we generally prefer to relax medication exclusions in our studies, with the goal of obtaining a large sample that includes patients both on *and* off medications. Given that neuroimaging study samples sizes are steadily increasing, the analysis of subgroups is more feasible. In a study of 40 individuals with depression, half of whom are taking antidepressants, analysis of subgroups can test for effects that are specific to medications. Of course, the power of subgroup analyses will depend on a number of factors, including subgroup sizes, the specific experimental questions being asked, and the number of different medication classes included. This approach must also address potential severity differences between subjects with and without medications. In addition to subgroup analyses, there are statistical strategies that (combined with appropriate sample selection) can account for medication effects, such as including medications as factors in an analysis of variance. Thus, we contend that by placing the burden on the analyses, recruiting samples of adequate size improves generalizability and clinical significance compared to excluding large groups of patients. If the goal of a research program is to understand a disordered population, our position is that inclusion criteria should be structured to capture as many exemplars of the disorder as possible, in order to understand how interacting variables are relevant to the phenotype and endophenotypes of the disorder.

### 2.4 Considerations for control groups and studies of typically developing children

Control populations are often not well characterized, especially in neuroimaging studies. Thus, there are many factors that may influence the results of studies that include typically developing children. The field has taken a step in the right direction, as many studies now estimate IQ. Yet, more extensive characterization is needed. Of course, it is not clear what information will be the most useful, and there exist a large number of measures that can be assessed. Thus, choices can be informed by the particular research questions and by the populations under study. In a study of typical development, such as those measuring growth curves of brain structure (e.g., Giedd et al., 1999, Sowell et al., 2004, Thompson et al., 2000), measures of pubertal status are often useful (e.g., Carskadon and Acebo, 1993), as hormones can influence brain development and age is not always an adequate proxy for pubertal stage. In studies in which a patient population is being compared to a control group, it is useful to assess clinical characteristics in the control group in addition to the patient group. Just because a control subject has never received a formal diagnosis does not mean that he/she does not have any neuropsychiatric problems. For example, many children may not meet diagnostic criteria for ADHD, but still have sub-clinical attention problems that influence daily life and hence, brain function. In fact, one large, careful epidemiological study of youth in rural America found that 21% of children in the population met criteria for one or more DSM-IV psychiatric disorders in the past three months, yet only 36% of those diagnosable children had received any mental health care in the same three-month period (Angold et al., 2002). Additionally, children without clinical diagnoses at the time of study may go on to develop diagnoses later in childhood or adolescence. Conversely, a child may have had past symptoms that went unnoticed or that parents do not recall. For example, a direct observational study of tics found at least one motor tic in 47% of first-graders, but in only 15% of 6th-graders (Snider et al., 2002). Most of the “missing” 32% in 6th grade would likely deny a history of tics, since no studies have found a prevalence of *chronic* tics greater than ∼10%. In these cases, was the child a valid control subject at the time of study? Of course, controlling for future development of neuropsychiatric illness or for past, unnoticed symptoms is quite difficult without life-long longitudinal study, which is not realistically feasible. Multiple-informant semi-standardized diagnostic interviews such as the Kiddie-Schedule for Affective Disorders and Schizophrenia (K-SADS) (Kaufman et al., 1997) can provide a reasonable surrogate, though at a substantial cost. In some cases, family history may be an appropriate proxy for past or future risk of disorder in children.

When designing a research study and defining inclusion/exclusion criteria for a control group, it is worth considering whether or not the control group should be excluded for medications and/or conditions commonly comorbid with the disorder under study. For example, a study of TS with liberal inclusion criteria for the patient group may consider whether or not it would be appropriate to apply the same criteria to the control group. Such decisions may depend on the scope of the study. A smaller-scale study with limited funds or a pilot study may benefit from excluding comorbidities and medications in the control group (regardless of the criteria for the patient group) in order to best differentiate patients and controls. On the other hand, this choice may confound interpretation of group differences. A large-scale or multisite study capable of recruiting large numbers of subjects may benefit from being inclusive and analyzing subgroups within the controls as well as within the patients. For instance, a well-matched control group for a TS study in which 40% of tic subjects have ADHD may well be a tic-free control group ∼40% of whom also have ADHD. This choice will largely depend on feasibility and the questions at hand. In any case, justification should be made for choices regarding inclusion/exclusion of control samples in addition to patient samples.

Just as one cannot assume that any particular patient group is homogeneous, nor can one assume that a control group is homogenous. In fact, there is evidence for behavior-based subgroups of typically developing children. Fair et al. (2012) demonstrated distinct subgroups of typically developing children based on neuropsychological performance, including measures of working memory, response inhibition, response variability, response speed, interference control, temporal information processing, and arousal and activation. Further, they found support for the idea that the heterogeneity in children with ADHD is nested within the normal variation shown in typically developing children. These results support the importance of collecting neuropsychological assessments from patients and controls alike. Interestingly, subgroups within typically developing children and children with ADHD can also be defined by fMRI data (Costa Dias et al., 2015). Thus, rather than comparing an entire group of children with ADHD to a group of controls, comparisons within subgroups may prove to be more informative.

### 2.5 Studying subjects who can hold still in the scanner

Neuroimaging researchers must also be aware of potential cohort effects specific to MRI studies. Some groups of subjects inevitably move more than others (e.g., children tend to move more than adults) and some neuropsychiatric conditions are even characterized by abnormal movement: TS involves tics, ADHD involves hyperactivity, Parkinson disease involves tremor, etc. Given the susceptibility of MRI data to motion artifact, which we discuss below, studies of these populations will necessarily be limited to those individuals who can hold still in the scanner. This issue brings up the question of whether or not we are capturing data that are truly representative of the population under study. In patient populations, conceivably only the least affected patients will be able to hold still during the scan. Interestingly though, in our experience with TS, some patients with clinically severe tics can hold quite still in the scanner, whereas some patients with clinically mild tics cannot hold still at all. Nevertheless, there may be a discrepancy between the amount of movement a subject exhibits on an average day and the amount of head movement present in the scans retained for analysis, in which case we may not be capturing data from the brain at the moments in time that are most typical for that subject. The sensitivity of MRI data to motion artifact renders these problems unavoidable. Since we know group differences in motion can introduce spurious results in MRI data (discussed below), our view is that matching head motion across groups is preferable to including subjects whose data are contaminated by motion. Other selection biases inherent in MRI studies include studying subjects who are not wearing orthodontic braces or who do not have claustrophobia. Of course, we must recognize and acknowledge these resulting cohort effects.

Furthermore, it is worth noting that there are a number of additional cohort effects that, while not specific to neuroimaging studies, nevertheless face neuroimaging researchers. For example, we can only scan subjects who are compliant, a concept that applies widely to human subjects research. Other unintentional selection biases could include living near a research center and having a parent with a flexible work schedule. When studying patient populations, there may be clinical features that differentiate groups other than the defining features of the disorder (e.g., lower IQ, sensory deficits). These cohort effects are also sometimes unavoidable, and partly why we advocate for inclusive, larger studies, in order to tease out those factors in post-hoc analyses.

## 3 Strategies for obtaining adequate data

Once a study sample has been selected, neuroimaging researches must strive to obtain useable imaging data. Collecting MRI data from pediatric and clinical populations comes with several challenges and has necessitated continual improvements in methods. The most common challenges are subject anxiety in the scanner environment, and head movement in the scanner (Poldrack et al., 2002, Bookheimer, 2000, Kotsoni et al., 2006). Failure to address these issues proactively can lead to loss of data due to aborted scans or excessive motion artifact. Additionally, the researcher loses valuable time, research funds, and other resources. Perhaps more importantly, inadequate attention to these issues can also produce misleading results (Power et al., 2012, Reuter et al., 2015, Yendiki et al., 2013, Le Bihan et al., 2006, Van Dijk et al., 2012, Satterthwaite et al., 2012). These problems also have ethical consequences, because if the research value to society is lower, so is the benefit:risk ratio for human subjects. Fortunately, several strategies can ameliorate these difficulties, though advancements in our methods are still needed. Herein we discuss the use and efficacy of current strategies.

### 3.1 Scanner environment

The MRI scanner environment will be unfamiliar to many research subjects, and this unfamiliarity will translate to varying levels of excitement and anxiety in each subject. Given the fundamental goal of acquiring adequate imaging data, the first step must be to ease this anxiety and make the experience as comfortable, pleasant, and child-friendly as possible. In addition, changes in subjects’ cognitive and affective states can affect brain activity and other physiological measures that influence imaging results, including respiratory rate, heart rate and muscle tension (e.g., Drevets et al., 1992). Moreover, if the subject is asked to return for longitudinal follow-up or is invited to participate in another study, a comfortable, pleasant experience will increase chances of continued participation. Successful developmental neuroimaging laboratories have taken several steps to minimize anxiety in the scanner. No one prescription will work for all children or special populations, and the experimenter must be flexible in response to the needs of each research participant.

Investigators generally agree that familiarizing children with the scanner environment before the scan itself is important. One strategy for familiarization is to show the child (and parent/guardian) a video that describes the MRI procedure in a child-friendly manner. Videos have been created by different groups, including Stanford University (http://cibsr.stanford.edu/participating/GettingReady/HomePreparation.html) and the University of Texas, Austin (https://www.youtube.com/watch?v=O6hQeqp5-EI&feature=em-share_video_user). Creating a video specific to your institution's MRI setup may be beneficial, though time consuming. Investigators can also offer at-home familiarization with the scanner sounds using audio recordings of several different scan sequences. Extensive protocols that use these and additional approaches have been developed to make scanning child-friendly. One example is the “submarine protocol” targeted toward 5- and 6-year-old children (Theys et al., 2014). Children embark on a “submarine adventure” consisting of extensive pre-scanning activities, including watching a video of “Whally the Whale,” popping bubbles with the researcher, crawling through a small tunnel while wearing ear plugs and headphones, and other engaging activities. For the MRI scan session, the scanner room is decorated as an underwater environment and the scanner itself is decorated as a submarine in order to continue the submarine theme. This protocol resulted in 95% of 5- and 6-year-old children completing the full scan session, suggesting success in easing anxiety.

A widely used method for familiarization with the scanner environment is the use of a mock scanner, which is a replica of the MRI scanner without the magnet. Commercial mock scanners often consist of a bore with a mechanically or manually operated bed and mock head coil. They may also include a mirror and back projection system or visual presentation goggles, simulating the real scanner environment. Audio recordings of scanner sounds can be played through speakers in the bore. Subjects can spend time in the mock scanner, becoming comfortable lying in the bore and listening to the scanner sounds. For particularly anxious children, we have taken the approach of putting the parent/guardian inside the mock scanner first, allowing the child to press the button that controls the scanner bed. The child can then take turns with his/her parent going inside the bore. Detailed mock scanner protocols have been published (de Bie et al., 2010, Raschle et al., 2009, Barnea-Goraly et al., 2014). Since commercial mock scanners involve significant cost, it may not be feasible for some institutions to acquire such a set-up. Fortunately, an inexpensive alternative practice set-up that uses a toy tunnel has been shown to work as well as a commercial mock scanner for obtaining T1- and diffusion-weighted scans (Barnea-Goraly et al., 2014).

During the MRI scan, there are strategies that can be implemented to create a child-friendly experience. For younger children, a fairly simple approach is to allow the child to bring a stuffed animal (MR compatible) into the scanner. Experimenters can also use a parent's presence to alleviate anxiety. However, the effects of a parent's presence can vary from child to child. Some children may be most comfortable with their parent in the scanner room, some may need to talk to their parent over the intercom, and some may be more comfortable without their parent in the MRI suite at all. Again, being attentive to the child's needs and being flexible in response to those needs is critical. Moreover, the experimenter must prospectively and consistently address safety issues involved in allowing parents into the scanner room. Another way to make the experience pleasant is to show the child a movie whenever possible. For every scan, we make sure that a movie or television show of the child's choice is cued and ready, and we begin playing the movie as soon as the child is inside the bore. Even if the child will only be watching the movie for a few minutes at a time, this strategy ensures that he/she will have something fun to watch while the experimenters finish setting up and run the initial scans (e.g., localizer). An additional tip that we have implemented for the especially anxious child is to explain that the first scan (the localizer) is very short (∼10 s), so they can see what a real scan is like in the amount of time it takes to count to ten in their head. In our experience, anxious children have responded well to this information. Some investigators opt to use shorter scan runs based on the assumption that shorter runs will be more tolerable for children. In calibrating the duration of resting-state fMRI runs from our experiences, we have found that 7-min runs are just as tractable as 5-min runs for most children that we have scanned. For those children who could not tolerate the scan and chose to stop, attempts at shorter runs did not seem to make a difference with respect to our ability to gather usable data.

### 3.2 The problem of head movement

Head movement in the scanner during structural and functional MRI data acquisition is known to disrupt the signal and cause artifacts. The assumption from the data analysis standpoint is that each voxel represents signal from one part of the brain. Unfortunately, even if the subject shifts his/her head by only half the width of a voxel (e.g., 2 mm), the data collected from each voxel will include signal from two different parts of the brain. At a minimum, motion will result in blurring of the image, which can reduce apparent volume, density or activity (Reuter et al., 2015, Smith and Nayak, 2010, Friston et al., 1996).

For fMRI data, motion will affect each voxel's measured blood oxygenation level dependent (BOLD) timecourse, reflecting signal from multiple voxels and altering signal intensity. There has been recent increasing interest in, and use of, functional connectivity MRI, which measures temporal correlations of BOLD signal between brain regions. Recently, it was discovered that this method comes with a very problematic, unforeseen motion artifact: small, sub-millimeter head movements systematically affect fMRI correlations (Power et al., 2012, Van Dijk et al., 2012, Satterthwaite et al., 2012). Specifically, correlations between spatially proximate voxels are inflated compared to correlations between spatially distant voxels. In other words, even very small, repeated head movements can introduce false correlations. For between-group analyses, this effect of motion can lead to spurious results when one group systematically moves more than the other. Thus, this issue is particularly critical to consider in studies that compare different age groups or patient populations, and has called into question previous functional connectivity findings in development and aging (e.g., Andrews-Hanna et al., 2007, Fair et al., 2007). There have also been recent discoveries that small head movements can lead to systematic artifacts in structural MRI measures, including volume and thickness measures (Reuter et al., 2015) and diffusion weighted imaging measures (Yendiki et al., 2013, Koldewyn et al., 2014). Thus, reducing head movement during data acquisition is critical for data quality and retention.

### 3.3 Methods to reduce head movement

There are a number of strategies that are commonly used in order to reduce the amount of head movement in the MRI scanner. Prior to the MRI scan, children can practice holding still while lying down. A group at Stanford University developed a “statue game” (http://cibsr.stanford.edu/participating/Games.html) that children can play at home, earning stickers for holding still (visually determined by a parent/guardian) for increasing lengths of time. Thus, children can practice holding still before even setting foot in the research environment. Experimenters have developed interesting and creative ways to continue this preparation during the study visit itself. For example, as part of the submarine protocol mentioned above, children practice holding still while balancing a piece of candy on their nose (Theys et al., 2014). Some groups also show children pictures of crisp and blurry images (brains, cats, etc.) to convey the consequences of movement and the importance of holding still (Theys et al., 2014, Raschle et al., 2009).

Most commonly, investigators use a mock scanner for movement training. Some mock scanners are equipped with real-time motion tracking, in which a sensor detects the child's head position. Using this technology, the child can receive online visual feedback of his/her head movement. Some software packages (e.g., MoTrak Head Motion Tracking System, Psychology Software Tools, Inc.) allow the subject to view a bulls-eye on a screen and a moving crosshair that represents his/her head position. Subjects can then practice keeping the crosshair in the center of the bulls-eye by holding still. Subjects can also be shown a movie that will pause if head movement exceeds a set threshold. Thus, children are able to acquire a sense for the sensitivity of the scanner to movement and can undergo some level of operant conditioning. One caveat should be mentioned regarding motion sensors that are placed on the forehead. We have found that in children with TS, most of whom have facial tics, the motion sensor is inaccurate when the child is ticcing. For example, the motion sensor will move when the child raises his/her eyebrows or scrunches his/her nose even if the child's cranium remains quite still. In these circumstances, motion training can be frustrating and likely ineffective. On the other hand, sniffing or small movements of the lips or tongue often cause detectable changes in the position of the brain.

While the usefulness of mock scanner training for minimizing motion is generally accepted, the empirical evidence for its effectiveness is limited. For example, fairly liberal thresholds, which may not effectively reduce motion-related effects, have been used to assess scan quality after training (de Bie et al., 2010, Epstein et al., 2007). Given the recent evidence that sub-millimeter movements are problematic for functional connectivity data, stricter criteria may be needed. Most striking is that we were unable to find any studies in the literature that included a proper experimental control group. Since testing the effectiveness of mock scanner training was not the primary aim of most studies reporting on mock scanning, all subjects received the training. Consequently, it is difficult to assess whether the success rates reported, e.g., 70–100% useable scans (de Bie et al., 2010, Barnea-Goraly et al., 2014) or decreased motion during actual vs. mock scan (Epstein et al., 2007), were attributable to mock scanner training. Thus, it remains an empirical question whether or not mock scanner movement training significantly improves movement during MRI scans, and if it does, what method or amount of training is sufficient.

During the actual scan, several measures can be taken to increase the chances of acquiring useable data. Physical restraints are sometimes used, including bite bars, masks, cushions around the head, vacuum packs, and tape across the forehead. Some of these options are more easily tolerated than others. For example, thermoplastic masks, which mold to the shape of the subject's face, are more comfortable than a bite bar, which requires clenching one's teeth on an object placed in the mouth. When these options are not available, cushions or vacuum packs can be placed around the subject's head to maintain head position. Placing tape across the subject's forehead is an easy option, which does not restrain head movement per se, but serves as instant sensory feedback if movement occurs. Again, however, we are unaware of controlled studies testing the efficacy of these methods. Furthermore, certain clinical populations (e.g., TS, autism) are exceptionally uncomfortable having something touching the face.

### 3.4 Methods to account for head movement

Despite best efforts to minimize head movement during data collection, the strategies just discussed do not completely eradicate motion in the data. Particularly when collecting data from developmental and clinical populations, it is nearly inevitable to have some degree of motion contamination. Thus, researchers have developed approaches to assess data quality and to account for motion in acquired data during processing and analysis.

For T1-weighted structural MRI scans, motion results in visible banding artifacts in the images and reduced gray matter/white matter contrast. Thus, researchers often adopt qualitative (visual grading of images) and/or quantitative criteria (errors in automated segmentation software, such as Freesurfer) by which to exclude subjects with too much artifact. For fMRI data, motion can be computed from realignment estimates, allowing investigators to obtain quantitative measures of movement in functional scans. Summary estimates have traditionally been used to assess which subjects and fMRI scan runs can be included in the data analysis. For example, an investigator might set a threshold for average motion at 1.5 mm, and any run with average motion exceeding 1.5 mm root mean square (rms) will be excluded from data analysis (as in Church et al., 2008). However, investigators must be aware of the different ways by which motion estimates can be calculated. Motion can be computed as absolute displacement from a reference frame (timepoint) or as relative displacement on a frame-by-frame basis, and different calculations can be applied to obtain the estimates (e.g., root-mean-square, summing absolute values). Thus, understanding the specific approach used in any given study is important, as the scales can differ across studies depending on the reference frame or calculations employed (Power et al., 2015).

Nearly every processing stream for fMRI data includes steps aimed at correcting head movement, including spatial realignment and regressing motion estimates. Given the discovery that sub-millimeter movements systematically affect correlations in fMRI signal timeseries, additional processing methods are needed to better account for motion artifact in functional connectivity data. Thus, there has been continued development of data processing methods to reduce these effects. For example, some investigators propose methods that include nuisance regressors, beyond those commonly used in functional connectivity preprocessing, that better account for local signal (Jo et al., 2013, Muschelli et al., 2014). Others have used group-level covariates of no interest, which are effective in reducing motion-related group differences, but also remove effects of interest that are collinear with the covariate (Satterthwaite et al., 2012). Our laboratory has devised and implemented a data preprocessing approach that involves volume censoring and global signal regression to minimize motion artifact (Power et al., 2014). Volume censoring involves excising from data analysis BOLD volumes with frame-by-frame movement (framewise displacement: FD) exceeding a strict threshold (e.g., 0.2 mm), thereby excluding movement-contaminated data points. While a consensus has not been reached as to which method works best, or even on how to measure success of these methods (for detailed assessment and review of multiple methods, see Power et al., 2015, Yan et al., 2013), there is no question at this point that ignoring head movement can produce artifactual groupwise functional connectivity results.

**Fig. 1:**
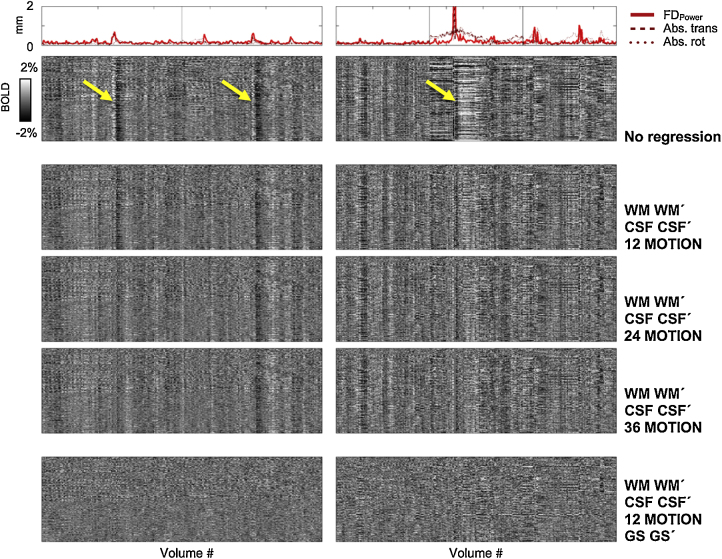
Pictorial display of resting state fMRI gray matter signals from 2 adult subjects. Top 2 panels show movement traces (top) and gray matter signal intensity (second from the top). The middle 3 gray panels show the effect of regressing increasing numbers of motions parameters. The bottom panel shows the effect of regressing the global signal. Yellow arrows point to examples of motion artifact that remain in the data until global signal regression is applied. FD_Power_: framewise displacement as calculated in Power et al. (2012); WM: white matter signal; CSF: ventricle signal; GS: global signal (whole-brain average); *R*: realignment estimates (6 parameters representing translation and rotation). The 12 motion parameters are [*R R*′], the 24 are [*R R*^2^*R*_*t*−1_*R*^2^_*t*−1_], and the 36 are [*R R*^2^*R*_*t*−1_*R*^2^_*t*−1_*R*_*t*−2_*R*^2^_*t*−2_], where subscripts represent the estimates from 1 or 2 previous frames, the prime represents the difference from the previous frame, and the superscript 2 means squared. Figure modified from Power et al. (2015). (For interpretation of the references to color in this figure legend, the reader is referred to the web version of this article.)

Whichever method one chooses to implement, we recommend careful examination of the data for residual artifacts. For example, visual inspection of individual subjects’ BOLD signal across time in gray matter voxels reveals visible artifact that is removed after global signal regression (Fig. 1)^1^. In addition, thresholds for movement criteria largely depend upon the data itself. While our laboratory uses an FD threshold of 0.2 mm for our 3T resting state fMRI dataset acquired using particular scan sequences, other datasets may require different thresholds. “A practical corollary is that if FD measures are to be used in some dataset, FD waveforms and magnitudes should be established (and reported) for that specific dataset, since magnitudes from the literature may not be appropriately scaled to the data at hand” (Power et al., 2015). Thus, it is recommended that researchers examine their data carefully and publish such examinations.

Compared to functional connectivity data, task fMRI data is less systematically affected by very low amplitude head movements. Of course, investigators ought to test whether motion correlates with task conditions (i.e., subjects move more during condition A than during condition B) in order to ensure that task effects are not driven by movement. Generally, sub-millimeter movements do not systematically affect groupwise results for task fMRI studies, making it less imperative to include additional processing steps like those used in functional connectivity studies. Interestingly though, task fMRI data quality can be substantially improved by more assiduous attention to these sub-millimeter amplitude head movements (Siegel et al., 2014).

In order to increase the odds of retaining useable data, we recommend collecting more data. Averaging multiple T1 weighted images, for example, often results in better estimates of structural measures. For task fMRI, the precise amount of data largely depends on the parameters of the task, but collecting additional runs may preserve a subject who would otherwise be excluded. For resting state fMRI, more data will reduce the likelihood of losing the subject entirely in the analysis, particularly when implementing processing methods that require removal of movement-contaminated data. There has been a prevailing idea that 5 min of resting state fMRI data was sufficient to measure resting state networks (Van Dijk et al., 2010), leading many groups to implement a practice of adding one 5-min resting state scan to the end of other scanning protocols. However, advances in the field and updated information now argue against this practice. For one, some of the most careful data processing methods to account for motion artifact require excising volumes of BOLD data (volume censoring, as discussed above). Therefore, if only 5 min are collected, it is likely that less than 5 min will survive stringent processing thresholds, particularly when scanning children and patient groups that tend to move more. In addition, there is evidence that 5 min of resting state data has only moderate reliability for an individual's functional connectivity profile (Van Dijk et al., 2010, Shehzad et al., 2009). For investigators interested in individual subject functional connectivity, 10–25 min of usable resting state data is recommended if one wants to obtain reproducible measurements (Anderson et al., 2011, Laumann et al., 2015), while more than 30 min may be required for more complex analyses with finer spatial specificity (Laumann et al., 2015). It is understandable that shorter scan runs and scan sessions are attractive when studying children and other special populations, and longer scan times may reduce a subject's ability to tolerate the scan. However, given its susceptibility to motion, fMRI data loss is inevitable, and losing subjects altogether is quite undesirable. It is more expensive to recruit and bring in additional subjects than to spend more time with each one. Thus, we encourage collecting more data while implementing strategies that can help with tolerability, such as taking brief breaks during scans and repeat scan days. Of course, future advances in image acquisition or data analysis may allow shorter acquisitions for certain analytic purposes (e.g., Miranda-Dominguez et al., 2014).

There has been increasing effort to develop MRI sequences that help reduce the adverse effects of motion. For structural MRI scans, a prospective motion correction technique was developed that implements spiral navigator scans and online motion tracking so that images tainted by movement can be reacquired (Brown et al., 2010, White et al., 2010). Use of this method results in better quality scans assessed by both qualitative (visual inspection of the images) and quantitative measures (fewer errors in Freesurfer cortical surface reconstruction and improved reliability of subcortical volume measurements). For functional MRI scans, the emergence and continued development of multi-echo sequences has sparked interest in the potential benefits these sequences may have for data quality (Kundu et al., 2012). By acquiring BOLD data at more than one timepoint during a single TR, the idea is that real signal can be more readily distinguished from artifactual signal. Multi-echo sequences may also allow computation of quantitative R2* images that may be more stable over time, addressing a key limitation of traditional BOLD imaging (Chen et al., 1996). However, it is presently unclear whether or not multi-echo sequences will significantly reduce motion artifacts (Power et al., 2015). Thus, methods development for improving MRI data acquisition (and analysis) is a continuing field of study. Given that developmental and clinical populations tend to move more than their common comparison groups, there is a paramount need for improved strategies for collecting high quality data.

## 4 Conclusions

Neuroimaging studies of developmental and clinical populations present several challenges and issues for consideration at the subject selection and data acquisition stage. Neuroimaging is expensive, amplifying the importance of obtaining the highest quality data, losing as little data as possible, and generating results that have the highest potential for real-world application. Of course, there are trade-offs that must be faced between the desire to have a well-sampled, deeply characterized dataset and the cost of acquiring such data in terms of both time and money. Funds and resources are not always available to collect multiple T1-weighted scans, 30 min of resting state fMRI data, a full battery of clinical and behavioral measures with multiple informants, family studies or thorough family history, etc. Subject factors, such as comfort and time commitment, can limit the extent of the data collection as well. Thus, weighing the many trade-offs can lead to difficult choices. We believe in two principled approaches to these decisions. (1) There is no point in having useless MRI data. Given the high cost involved in neuroimaging and the susceptibility to movement artifact, every attempt to collect useful (which sometimes translates to “enough”) MRI data should be employed. In our experiences with children with TS, collecting two 5-min resting state fMRI scans resulted in retaining only 50% of subjects due to movement, while collecting three to four 7-min scans resulted in retaining 75% of subjects. Collecting more data from each individual yielded significantly less wasted data, which we consider well worth the up-front investment. (2) Omitting phenotyping altogether is, as the saying goes, “penny-wise and pound-foolish.” Tough choices may need to be made with regard to the extent of phenotyping, but some level of subject characterization is necessary in each of the most important phenotypic domains for the population being studied. For instance, several neuroimaging findings in studies of TS have proven to be best interpreted only together with information on comorbid ADHD (Castellanos et al., 1996, Gilbert et al., 2004). Depending on its specific aims, a TS study may require only a brief estimate of current ADHD symptom severity or it may require expert assessment of lifetime ADHD diagnosis, but ignoring ADHD altogether would be hard to justify. Again, it is well worth the up-front investment to characterize the sample in order to obtain more interpretable results.

Addressing the issues involved in subject selection and obtaining adequate data is far from simple. As researchers, we must carefully and thoughtfully justify our choices of methods and study design. We should remain explicitly aware of our ultimate research goals and become better equipped to justify our decisions in light of those goals. As reviewers and critical readers of science, we must also have thoughtful rationale for our critiques of study designs and results. Importantly, we must be able to communicate this reasoning clearly to peers and colleagues. Here, we argue that generalizability of results is a crucially important consideration, as researchers should not strive for findings that hold true only under particular circumstances, but rather for findings that emerge regardless of methodological detail. Understanding the unintended consequences of our methodological choices and clearly communicating the rationale for these choices will lead to better science.

## Footnotes

1 Reprinted from Neuroimage, vol. 105, Power JD, Schlaggar BL, Petersen SE, Recent progress and outstanding issues in motion correction in resting state fMRI, pg. 536–551 (2015), with permission from Elsevier.
